# Microarray Analysis of Gene Expression Provides New Insights Into Denervation-Induced Skeletal Muscle Atrophy

**DOI:** 10.3389/fphys.2019.01298

**Published:** 2019-10-11

**Authors:** Yuntian Shen, Ru Zhang, Liang Xu, Qiuxian Wan, Jianwei Zhu, Jing Gu, Ziwei Huang, Wenjing Ma, Mi Shen, Fei Ding, Hualin Sun

**Affiliations:** ^1^Key Laboratory of Neuroregeneration of Jiangsu, Ministry of Education, Jiangsu Clinical Medicine Center of Tissue Engineering and Nerve Injury Repair, Co-Innovation Center of Neuroregeneration, Nantong University, Nantong, China; ^2^The Second Affiliated Hospital of Nantong University, Nantong University, Nantong, China; ^3^Department of Surgery, Changshu Affiliated Hospital of Nanjing University of Chinese Medicine, Changshu Traditional Chinese Medicine Hospital, Changshu, China; ^4^Department of Medical Laboratory, School of Public Health, Nantong University, Nantong, China; ^5^Department of Orthopedics, Affiliated Hospital of Nantong University, Nantong, China

**Keywords:** denervation, skeletal muscle atrophy, microarray, bioinformatics, oxidative stress, inflammation

## Abstract

Denervation induces skeletal muscle atrophy, accompanied by complex biochemical and physiological changes, with potentially devastating outcomes even an increased mortality. Currently, however, there remains a paucity of effective strategies to treat skeletal muscle atrophy. Therefore, it is required to understand the molecular mechanisms of skeletal muscle atrophy and formulate new treatment strategies. In this study, we investigated the transcriptional profile of denervated skeletal muscle after peripheral nerve injury in rats. The cDNA microarray analysis showed that a huge number of genes in tibialis anterior (TA) muscles were differentially expressed at different times after sciatic nerve transection. Notably, the 24 h of denervation might be a critical time point for triggering TA muscle atrophy. According to the data from self-organizing map (SOM), Pearson correlation heatmap, principal component analysis (PCA), and hierarchical clustering analysis, three nodal transitions in gene expression profile of the denervated TA muscle might partition the period of 0.25 h–28 days post nerve injury into four distinct transcriptional phases. Moreover, the four transcriptional phases were designated as “oxidative stress stage”, “inflammation stage”, “atrophy stage” and “atrophic fibrosis stage”, respectively, which was concluded from Kyoto Encyclopedia of Genes and Genomes (KEGG) and Gene ontology (GO) analyses at each transcriptional phase. Importantly, the differentially expressed genes at 24 h post sciatic nerve transection seemed to be mainly involved in inflammation, which might be a critical process in denervation-induced muscle atrophy. Overall, our study would contribute to the understanding of molecular aspects of denervation-induced muscle atrophy, and may also provide a new insight into the time window for targeted therapy.

## Introduction

Skeletal muscle, as a highly plastic organ, is generally capable of adapting to mild external changes through secretion of myokines and myometabolites, thus maintaining whole body homeostasis (Aversa et al., 2019; Oost et al., 2019). However, a diverse array of pathological stimuli, including disuse, nutrition deprivation, denervation, and systematic disease (e.g., AIDS, sepsis, renal and cardiac failure, muscular dystrophies, or cancer), are likely to induce skeletal muscle atrophy, characterized by muscle weakness and dysfunction (Bonaldo and Sandri, 2013; Szentesi et al., 2019). Skeletal muscle atrophy may cause adverse impacts on the quality of life and life prognosis in patients. Unfortunately, currently available treatments are often unable to achieve desirable therapeutic outcomes (Qiu et al., 2018). In this sense, it is urgently required to better understand molecular mechanisms of skeletal muscle atrophy and actively seek new therapeutic strategies.

During skeletal muscle atrophy, a series of biochemical and physiological alterations appear in atrophic muscle, triggering the gene expression changes. Bodine et al. (2001) and Bodine and Baehr (2014) demonstrate that muscle RING finger 1 (MuRF1) and muscle atrophy F-box (MAFbx), two muscle-specific E3 ubiquitin ligases, are up-regulated in different models of muscle atrophy, playing a pivotal role in atrophy programs; Wu et al. (2014) indicate that NF-kB sites are necessary for MuRF1 promoter activation in disuse-induced muscle atrophy. Recently, an increasing number of studies have adopted cDNA microarray to reveal that many genes are differentially expressed in atrophic muscles (Gomes et al., 2001; Kunkel et al., 2011; Fedorov et al., 2014; Kruger et al., 2015; Jeong et al., 2017; Weng et al., 2018). Although these findings provide extensive gene expression data, their design of time points is not comprehensive enough to fully cover the entire process of muscle atrophy, especially not including an early stage. A global analysis is necessary for obtaining more valuable information on gene expression profiles of skeletal muscle in response to different atrophy-inducing stimuli.

Animals with sciatic nerve transection have been commonly chosen as a study model for denervation-induced skeletal muscle atrophy (Madaro et al., 2018; Lala-Tabbert et al., 2019). Once sciatic nerve is transected, target muscles will lose their function of “muscle pump” due to the loss of nerve innervation, which leads to relatively reduced perfusion of target muscles, facilitating skeletal muscle atrophy (Laughlin, 1987; Marra et al., 2015). This type of skeletal muscle atrophy is regulated by different molecular mediators (Tang et al., 2014; Li et al., 2017; Choi et al., 2018; Pigna et al., 2018; Lala-Tabbert et al., 2019), but the issue of how molecular mediators are deliberately orchestrated to activate atrophic programs is to be systematically addressed. The purpose of this study was to furnish a global perspective of transcriptional regulation for denervation-induced skeletal muscle atrophy. We constructed a model of denervated skeletal muscle atrophy by using the tibialis anterior (TA) muscles of rats which underwent sciatic nerve transection, and performed cDNA microarray to identified thousands of differentially expressed genes (DEGs) in TA muscles at different times after sciatic nerve transection. The microarray data were then analyzed by clustering and bioinformatic methods to visualize some interesting aspects of gene regulation in atrophic skeletal muscles during the denervation period.

## Materials and Methods

### Animal Experiment

A total of 72 adult male Sprague-Dawley (SD) rats (weighing ∼200 g) were provided by the institutional Experimental Animal Center (Nantong, China), and were maintained under temperature 22°C and a 12-h light/dark cycle with free access to food and water. The experiments involving animals were carried out in accordance with the animal care guidelines of Nantong University and ethically approved by Jiangsu Administration Committee of Experimental Animals. The animals were randomly divided into 11 experimental groups and a sham group (*n* = 6 each group). For experimental groups, the rats were anesthetized with intraperitoneal injection of mixed narcotics (100 mg/kg ketamine plus 10 mg/kg xylazine) before the sciatic nerve was exposed through an incision at the mid-thigh of the left hind limb for transection to leave a 10-mm long defect. For relieving post-surgery pain, the rats were given buprenorphine (0.2 mg/kg subcutaneously) when performing the operation. The rats were sacrificed by cervical decapitation to harvest TA muscles at 0.25, 0.5, 3, 6, 12, and 24 h, 3, 7, 14, 21, and 28 days post nerve injury respectively. For a sham group, the rats were subjected to similar surgical procedures but without sciatic nerve transection, and TA muscles were harvested immediately after rat sacrifice (referred to as at 0 h post surgery).

### Microarray Analysis

The TA muscle sample was homogenized, and RNA was extracted with RNasey Mini Kit (Qiagen, San Francisco, CA, United States) as per kit guidelines. The quantity of total RNA was measured by using NanoDrop (ND-2000 Thermo Scientific, Delaware, ME, United States), and RNA integrity was evaluated by using Agilent Bioanalyzer 2100 (Agilent Technologies, Santa Clara, CA, United States). Microarray analysis consisted of several successive steps, including sample labeling, hybridization, scanning, and image analysis, according to standard protocols. In brief, total RNA was transcribed to double strand cDNA and synthesized into cRNA. After labeled with Cyanine-3-CTP (Cy3), the cRNA was hybridized onto Agilent SurePrint G3 Rat GE microarray (8 × 60,000). Agilent Scanner (G2505C) was used to scan microarray slides, and Agilent Feature Extraction Software (version 10.7.1.1) was used to read the scanned images. The raw data were quantile-normalized and log2 transformed for processing with Genespring Software (version13.1). The probes for which at least 60% samples have flags in “Detected” were chosen for further data analysis.

The expression level of mRNAs at each time was compared to that at 0 h post surgery (control). The gene whose mRNA expression showed a fold change of greater or lower than 2 and a *P*-value of less than 0.05 was considered a DEG.

### Time-Specific Pattern Identification

Microarray data mining was performed by a combination of self-organizing map (SOM), Pearson correlation heatmap, principal component analysis (PCA), and hierarchical clustering analysis to identify time-specific patterns in the mRNA expression of DEGs.

SOMs, as a class of unsupervised neural networks, is used to build up 1-, 2-, or 3-dimensional representation of high dimensional information (Chavez-Alvarez et al., 2014). Here SOMs were created by using Matlab software R2008a (MathWorks, Natick, MA, United States). Pearson correlation coefficients were calculated by the cor function of R “stats’ package^1^ for ensuring the reliability of expression data, and the results were viewed as a heatmap by ‘pheatmap’ R package (Kolde, 2015). PCA is an unsupervised learning method for constructing high-dimension data patterns without reference to prior knowledge, and the transformation in PCA is achieved by geometrically projecting data onto special dimensions called principal components (PCs) (Lever et al., 2017). Here PCA were performed by R ‘prcomp’ function of R ‘stats’ package^1^, where the first 3 PCs were selected to view the expression pattern of samples. Hierarchical clustering analysis was performed from Euclidean distance matrix data by using the complete-linkage cluster in the R ‘dendextend’ package (Galili, 2015), and Euclidean distance measure was used to calculate the similarity in gene expressions between samples, and to group samples into clusters by the Ward.D method in the R ‘stats’ package^1^.

### Functional Annotation

Kyoto Encyclopedia of Genes and Genomes (KEGG) database (Release 91.0) and Gene Ontology (GO) category database were applied for functional annotation of DEGs. KEGG pathway enrichment analysis was tested upon hypergeometric distribution by R ‘phyper’ function (Khatri et al., 2012). Enrichment analysis of GO categories was performed by DAVID tools^2^. Up- or down- regulated genes were separated for functional enrichment analysis. KEGG or GO categories with a *P-*value of less than 0.05 were considered the significantly enriched ones, which were viewed in a heatmap. The expression profile for each enriched category was shown based on the average *z*-score transformed expression value. *Z* score (x) = (x−μ)/δ, where μ is the mean value and δ is the corresponding standard deviation (Parikh et al., 2010).

### Quantitative Real Time PCR (qRT-PCR)

For qRT-PCR validation, the primers, designed by Primer Premier 5.0 software (Premier Biosoft International, Palo Alto, CA, United States), were purchased from Generay Biotech (Shanghai, China). They are listed in Supplementary Table S1. The RNA samples were reverse transcribed and synthesized to cDNA with an iScript cDNA synthesis kit as per the kit manual (Bio-Rad, Hercules, CA, United States). The qRT-PCR reaction was conducted on an ABI7500 Real Time System (Applied Biosystems, Foster City, CA, United States) by using SYBR Green I mix (Roche, Lewes, United Kingdom). The thermal cycle program was as follows: denaturation at 95°C for 10 s, annealing at 60°C for 30 s, extension at 70°C for 10 s (He et al., 2016). All reactions were performed in triplicates, and the specificity of PCR amplification was determined by melting point curve analysis. Quantification of the mRNA expression was performed on a 7500 real-time PCR system (Applied Biosystems). After the cycle threshold (Ct) values were determined based on the cycle number of PCR at which a threshold in fluorescence emission reached, the relative mRNA expression was calculated using comparative 2^–Δ^ ^Δ^ ^Ct^ method (Livak and Schmittgen, 2001), where beta actin (ACTB) was used as an internal control.

## Results

### Differential Expression of Genes in Denervated TA Muscles

**FIGURE 1:**
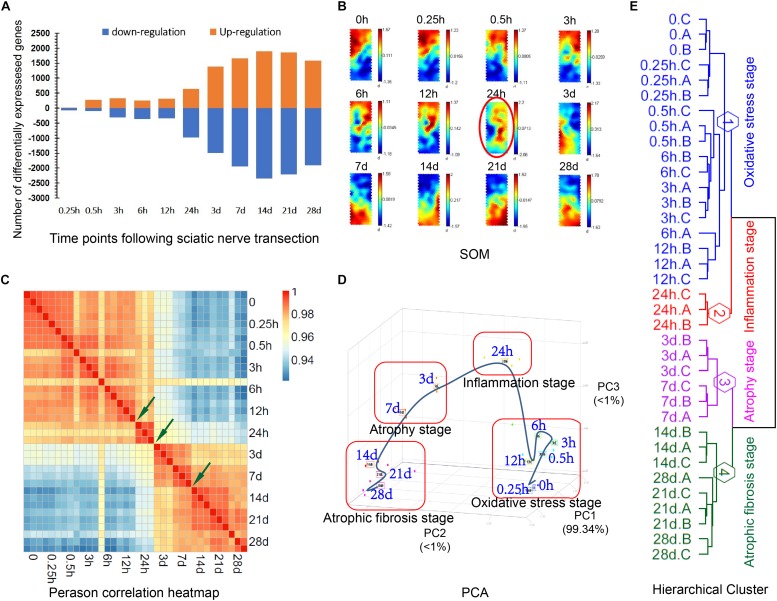
Four distinct transcriptional phases during denervation-induced muscle atrophy. **(A)** The number of DEGs at different times in TA muscles post nerve injury as compared to control (0 h post sham surgery). **(B)** SOMs: Color-coded output maps representing the final weight of denervated muscle atrophy. The cluster centroid value is coded with a range of colors from blue (for the lowest expression level) to red (for the highest expression level). **(C)** Pearson correlation heatmap between different times post nerve injury, as labeled on down and right margins. Arrows indicate three nodal transitions. **(D)** PCA of DEGs at different times post nerve injury. The percentage of variance captured by principal components (PCs) is shown. **(E)** Dendrogram of gene expression profiles, created by hierarchical clustering analysis. The three nodal transitions in temporal gene expressions segregate the four distinct transcriptional phases within the period of 28 days post nerve injury, and they were designated as “oxidative stress stage,” “inflammation stage,” “atrophy stage” and “atrophic fibrosis stage,” respectively.

The RNA samples were extracted from TA muscles and were subjected to cDNA microarray analysis. In each assay, at least three biologically independent samples were used to make data reproducible. A huge number of genes in experimental groups were differentially expressed as compared to those in control (Supplementary Table S2). Out of the DEGs, 14, 282, 327, 255, 310, 641, 1383, 1661, 1891, 1854, and 1578 genes were up-regulated, and 69, 104, 317, 366, 341, 976, 1488, 1951, 2348, 2212, and 1905 genes were down-regulated at 0.25, 0.5, 3, 6, 12, and 24 h, 3, 7, 14, 21, and 28 days post nerve injury, respectively (Figure 1A). Interestingly, the number of DEGs displayed a marked increase at 24 h post nerve injury, suggesting that the atrophic process might be extensively activated at this time point.

Based on SOM analysis, the gene expression profile in TA muscles might be considered to be composed of three main portions: (I) covering control (0 h), 0.25, 0.5, 3, 6 and 12 h; (II) covering 24 h; (III) covering 3, 7, 14, 21, and 28 days (Figure 1B), implying that 24 h post nerve injury was a critical time point in gene expression changes. Pearson correlation heatmap of microarray data indicated that three nodal transitions between 12 and 24 h, 24 h and 3 days, and 7 and 14 days post nerve injury were observable in the expression profile of DEGs (Figure 1C). PCA suggested that the period of 28 days post nerve injury could be partitioned into four distinct transcriptional phases by taking the three nodal transitions as boundaries between phases (Figure 1D). A dendrogram, created by hierarchical cluster (Figure 1E), provided further evidence for the existence of four transcriptional phases.

### KEGG Pathway Enrichment Analysis

**FIGURE 2:**
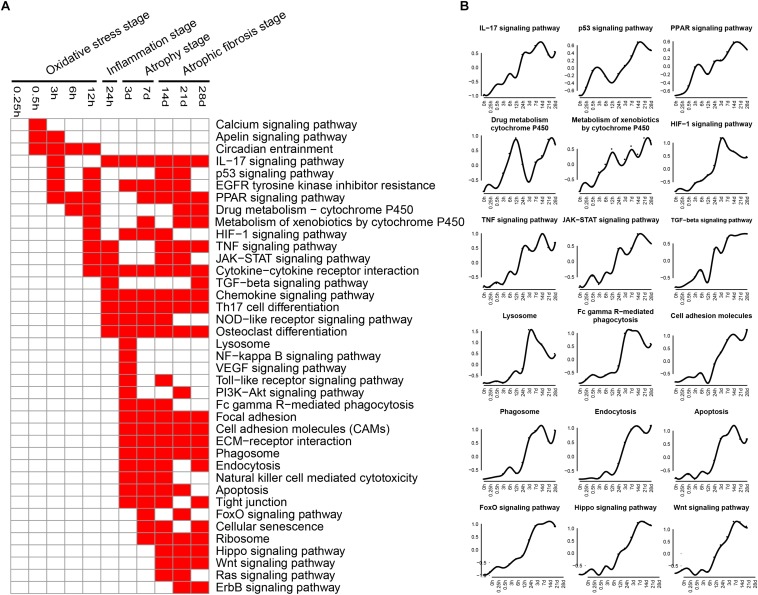
KEGG pathway analysis of up-regulated expressed genes in TA muscles after sciatic nerve transection. **(A)** The enriched categories (red color) of pathways at different times post nerve injury. **(B)** The average expression profiles of DEGs involved in major pathways. The *y*-axis represents the averaged *z*-score of genes in the specific KEGG pathway.

KEGG pathways related to DEGs were identified by using KEGG database. The enriched categories of KEGG pathways for the up-regulated genes at different times after sciatic nerve transection were labeled in Figure 2. During transcriptional phase I (0.25–12 h post nerve injury), the pathways, such as IL-17 signaling pathway, p53 signaling pathway, PPAR signaling pathway, drug metabolism-cytochrome P450, metabolism of xenobiotics by cytochrome P450, and HIF-1 signaling pathway, were enriched; also enriched were calcium signaling pathway, apelin signaling pathway, circadian entrainment, and so on. The data suggested that oxidative stress was mainly activated at this stage. During transcriptional phase II (24 h post nerve injury), the pathways, such as IL-17 signaling pathway, TNF signaling pathway, JAK-STAT signaling pathway, cytokine-cytokine receptor interaction, TGF-beta signaling pathway, chemokine signaling pathway, and NOD-like receptor signaling pathway, were all enriched, suggesting that inflammation was widely activated at this stage. During transcriptional phase III and IV (3–28 days post nerve injury), the pathways, such as lysosome, Fc gamma R-mediated phagocytosis, phagosome, endocytosis, apoptosis, FoxO signaling pathway, cell adhesion molecules (CAMs), extracellular matrix (ECM)-receptor interaction, cellular senescence, hippo signaling and Wnt signaling pathway, were enriched, suggesting that muscle atrophy and fibrosis were activated at these two stages.

**FIGURE 3:**
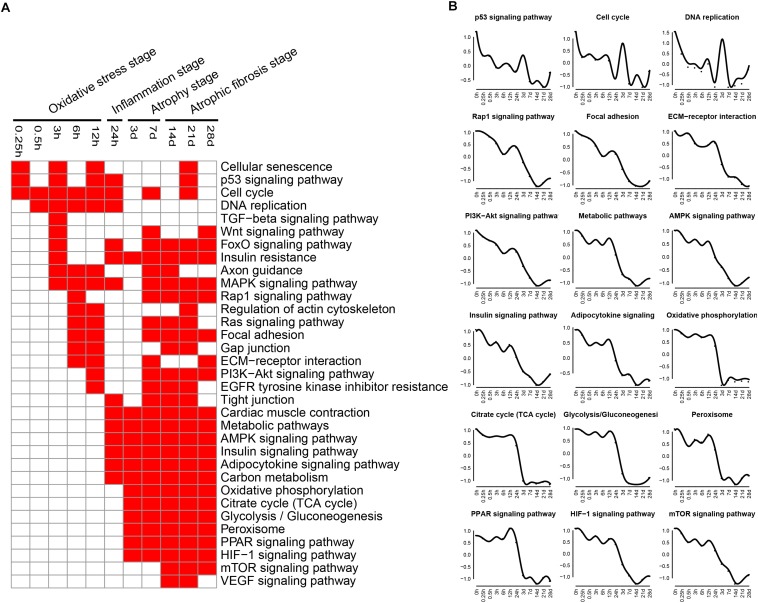
KEGG pathway analysis of down-regulated expressed genes in TA muscles after sciatic nerve transection. **(A)** The enriched categories (red color) of pathways at different times post nerve injury. **(B)** The average expression profiles of DEGs involved in major pathways. The *y*-axis represents the averaged *z*-score of genes in the specific KEGG pathway.

The enriched categories of KEGG pathways for the down-regulated genes at different times after sciatic nerve transection were labeled in Figure 3. During transcriptional phase I, the pathways, such as cell cycle, DNA replication, rap1 signaling pathway, TGF-beta signaling pathway, insulin resistance, MAPK signaling pathway and Ras signaling pathway, were enriched, suggesting that proliferation was inhibited and oxidative stress was activated at this stage. During transcriptional phase II, the pathways, such as MAPK signaling pathway, AMPK signaling pathway, insulin signaling pathway and adipocytokine signaling pathway, were enriched. During transcriptional phases III and IV, the pathways, such as metabolic pathway, oxidative phosphorylation, citrate cycle, glycolysis were enriched; also enriched were MAPK signaling pathway, AMPK signaling pathway, insulin signaling pathway, adipocytokine signaling pathway, Rap1 signaling pathway, PI3K-AKT signaling pathway, gap junction, and so on. These results collectively suggested that energy metabolism was significantly inhibited at the last three stages.

### GO Biological Process Analysis

**FIGURE 4:**
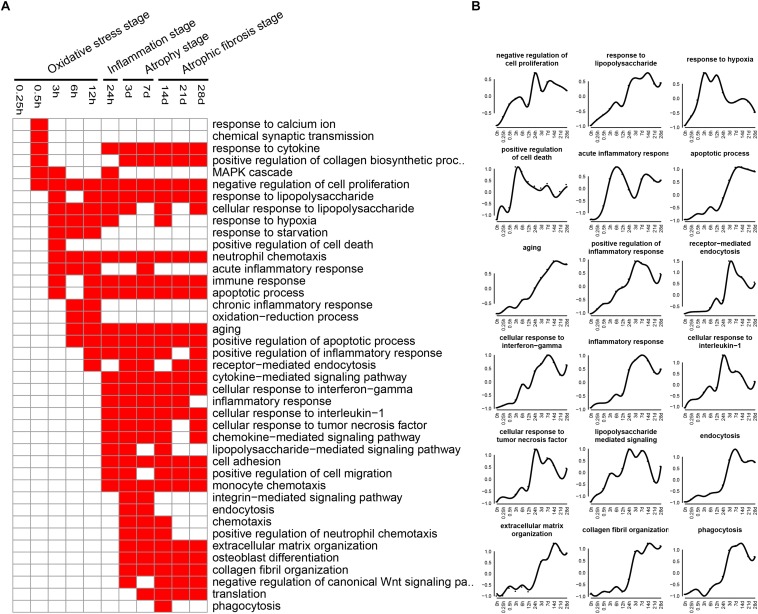
GO biological process analysis of up-regulated expressed genes in TA muscles after sciatic nerve transection. **(A)** The enriched categories (red color) of biological processes at different times post nerve injury. **(B)** The average expression profiles of DEGs involved in major biological processes. The *y*-axis represents the averaged *z*-score of genes in the specific biological process terms.

Biological processes related to DEGs were identified using GO analysis. The enriched categories of biological processes for the up-regulated genes at different times after sciatic nerve transection were labeled in Figure 4. During transcriptional phase I, the categories, such as response to calcium ion, response to cytokine, response to hypoxia, and oxidative-reduction process, were enriched; also enriched were MAPK cascade, negative regulation of cell proliferation, and positive regulation of cell death, and so on. These data suggested that stress response progress was activated at this stage. During transcriptional phase II, the categories, such as response to lipopolysaccharide, neutrophil chemotaxis, immune response, positive regulation of inflammatory response, inflammatory response, cellular response to interleukin-1, cellular response to tumor necrosis factor, and lipopolysaccharide-mediated signaling pathway, were enriched, suggesting that inflammatory response was widely activated at this stage. During transcriptional phase III and IV, the categories, such as endocytosis, phagocytosis, extracellular matrix organization, and collagen fibril organization, were enriched, suggesting that biological processes of muscle atrophy and fibrosis were activated at these two stages.

**FIGURE 5:**
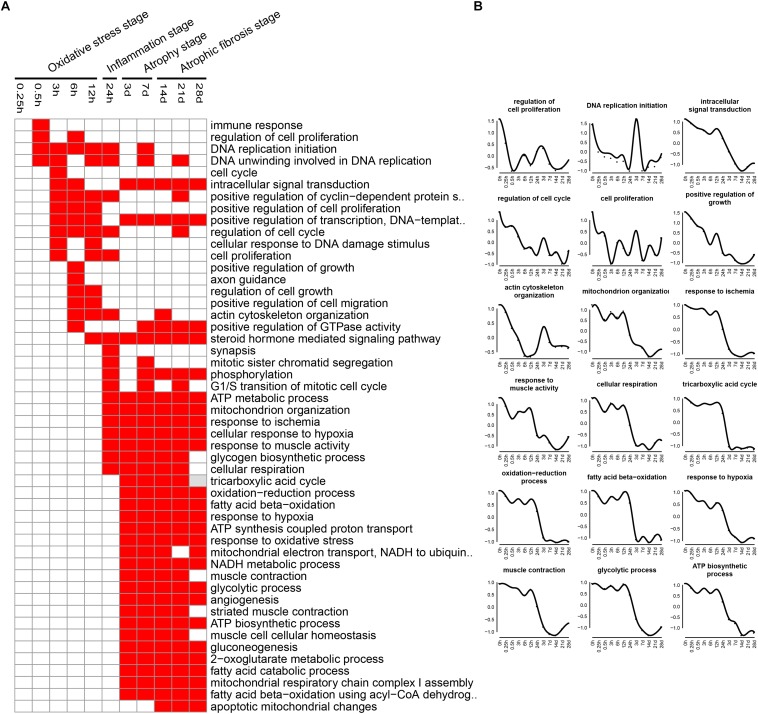
GO biological process analysis of down-regulated expressed genes in TA muscles after sciatic nerve transection. **(A)** The enriched categories (red color) of biological processes at different times post nerve injury. **(B)** The average expression profiles of DEGs involved in major biological processes. The *y*-axis represents the averaged *z*-score of genes in the specific biological process terms.

The enriched categories of biological processes for the down-regulated genes at different times after sciatic nerve transection were labeled in Figure 5. During transcriptional phase I, the categories, such as regulation of cell proliferation, DNA replication, cell cycle, positive regulation of cell proliferation, regulation of cell cycle, cell proliferation and positive regulation of growth, were enriched, suggesting that the cell proliferation was suppressed at this stage. During transcriptional phase II, the categories, such as response to ischemia, cellular response to hypoxia, mitochondrion organization, and ATP metabolic process, were enriched. During transcriptional phases III and IV, the categories, such as cellular respiration, tricarboxylic acid cycle (TCA cycle), fatty acid beta-oxidation, response to oxidative stress, and glycolytic process, were enriched. These results collectively indicated that biological processes of energy metabolism were significantly suppressed at the last three stages.

### GO Molecular Function Analysis

**FIGURE 6:**
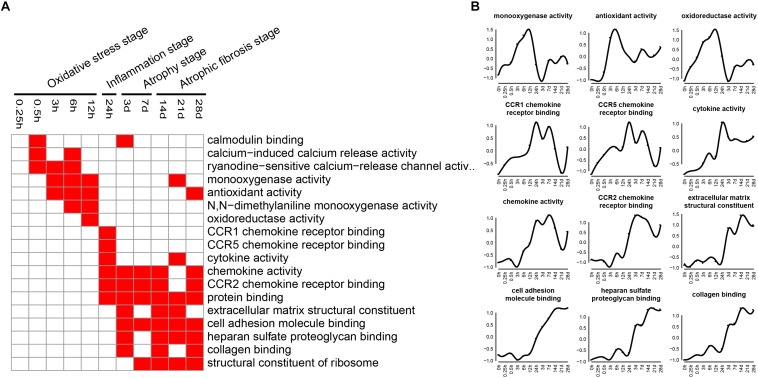
GO molecular function analysis of up-regulated expressed genes in TA muscles after sciatic nerve transection. **(A)** The enriched categories (red color) of molecular functions at different times post nerve injury. **(B)** The average expression profiles of DEGs involved in major molecular functions. The *y*-axis represents the averaged *z*-score of genes in the specific molecular function terms.

Molecular functions related to DEGs were also examined by GO analysis, and the enriched categories of molecular functions for the up-regulated genes at different times after sciatic nerve transection were labeled in Figure 6. During transcriptional phase I, the categories, such as calcium-induced calcium release activity, monooxygenase activity, *N,N*-dimethylaniline monooxygenase activity, and oxidoreductase activity, were enriched, suggesting that oxygenase activity was significantly enhanced at this stage. During transcriptional phase II, the categories, such as CCR1 chemokine receptor binding, CCR5 chemokine receptor binding, cytokine activity, chemokine activity and CCR2 chemokine receptor binding, were enriched, suggesting that inflammatory factor activity was significantly increased at this stage. During transcriptional phases III and IV, the categories, such as extracellular matrix structural constituent, cell adhesion molecule binding, heparin sulfate proteoglycan binding, collagen binding, and structural constituent of ribosome, were enriched, suggesting that structural components of ECM were significantly changed at these two stages.

**FIGURE 7:**
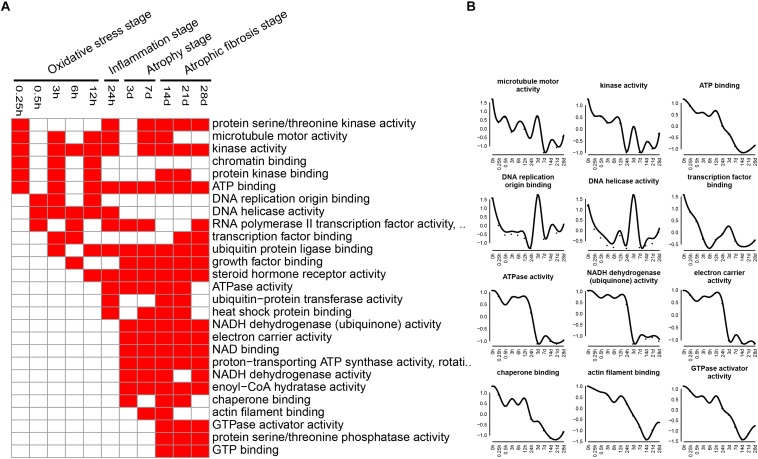
GO molecular function analysis of down-regulated expressed genes in TA muscles after sciatic nerve transection. **(A)** The enriched categories (red color) of molecular functions at different times post nerve injury. **(B)** The average expression profiles of DEGs involved in major molecular functions. The *y*-axis represents the averaged *z*-score of genes in the specific molecular function terms.

The enriched categories of molecular functions for the down-regulated genes at different times after sciatic nerve transection were labeled in Figure 7. During transcriptional phase I, the categories, such as microtubule motor activity, kinase activity, DNA replication origin binding, DNA helicase activity, RNA polymerase ii transcription factor activity, and transcription factor binding, were enriched, suggesting that the activities of DNA replication and transcription were reduced at this stage. During transcriptional phase II, the categories, such as ATP binding, ubiquitin-protein transferase activity, heat shock protein binding, and steroid hormone receptor activity, were enriched. During transcriptional phases III and IV, the categories, such as NADH dehydrogenase activity, electron carrier activity, chaperone binding, and GTPase activator activity, were enriched. These results collectively indicated that the energy metabolism was significantly inhibited at the last three stages.

### Four Transcriptional Phases

Based on KEGG and GO analyses, oxidative stress was activated and cell proliferation was inhibited mainly during transcriptional phase I; inflammation was widely activated during transcriptional phase II; muscle atrophy (phagosome, endocytosis, and lysosome) was activated and energy metabolism was significantly suppressed during transcriptional phase III; muscle atrophy and fibrosis (structural components of extracellular matrix) was activated during transcriptional phase IV. In this way, transcriptional phases I-IV might be sequentially referred to as “oxidative stress stage,” “inflammation stage,” “atrophy stage” and “atrophic fibrosis stage” to concisely specify major functions related to DEGs at different time stages.

### qRT-PCR Validation

**FIGURE 8:**
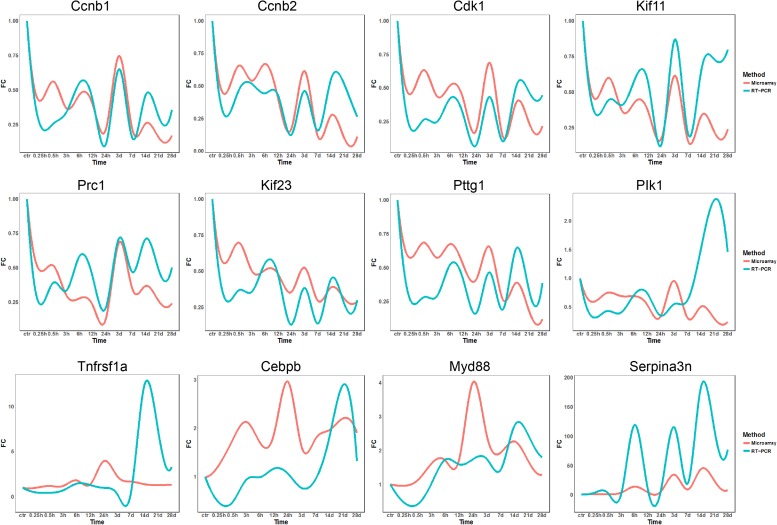
qRT-PCR validation of expression levels of DEGs in TA muscles after sciatic nerve transection. The curve plots comparing qRT-PCR and microarray data on the time-dependent expression changes of 12 genes.

To validate microarray data, 12 genes, including Ccnb1, Ccnb2, Cdk1, Kif11, Prc1, Kif23, Pttg1, Plk1, Tnfrsf1a, Cebpb, Myd88, and Serpina3, were subjected to qRT-PCR detection for their differential expressions. As to Ccnb1, Ccnb2, CDK1, Kif11, Prc1, Kif23, and Pttg1, qRT-PCR data were quite consistent with microarray results. As to other five genes, qRT-PCR data were roughly consistent with microarray results as far as the change tendency of gene expression was concerned, despite more or less deviations occurring at some time points (Figure 8). In general, however, qRT-PCR and microarray data were correlated to each other in this study.

## Discussion

Various pathological stimuli provoke skeletal muscle atrophy, which, in its nature, results from an imbalance of protein synthesis and protein degradation. The orchestrated cellular events and complex interactions between signaling pathways, mediated by an enormous number of functional biomolecules, are critically involved in the action mechanisms underlying skeletal muscle atrophy (Bonaldo and Sandri, 2013; Cohen et al., 2015; Bilodeau et al., 2016). Setting out from individual genes or proteins has proven to be a disappointing approach to acquiring a global knowledge about the mechanisms of skeletal muscle atrophy. In recent years, therefore, many studies have examined a large number of DEGs in atrophic muscles by microarray analysis (Gomes et al., 2001; Kunkel et al., 2011; Fedorov et al., 2014; Kruger et al., 2015; Jeong et al., 2017; Weng et al., 2018). Despite high-throughput data obtained in these pioneering studies, this study focused on microarray analysis over a broader time window of denervated skeletal muscle atrophy, and applied various clustering methods and bioinformatic tools to mine microarray data with a hope to obtain the detailed mechanistic information on denervated skeletal muscle atrophy.

We identified a huge number of DEGs in TA muscles after sciatic nerve transection. The number of either up-regulated genes or down-regulated genes in TA muscles showed a time-dependent change during the period of 0.25 h to 28 days post nerve injury. Interestingly, the number of DEGs displays a marked increase at 24 h post nerve injury, and SOM analysis also indicated that 24 h post nerve injury was a critical time point of the gene expression pattern in TA muscles, and that the most number of genes began to be activated at this time point, thus initiating the atrophic process.

Pearson correlation heatmap, PCA, and hierarchical cluster analysis were combined to analyze the gene expression profile in denervated TA muscles. The four transcriptional phases were divided within the period of 28 days post nerve injury, and different transcriptional phases might represent the activation of different functional genes, different signaling pathways, different biological processes, and different molecular functions (Sacheck et al., 2007; Glass and Roubenoff, 2010). This division provided a scientific basis for specific therapeutic interventions at different time stages of denervated skeletal muscle atrophy.

KEGG analysis and GO annotation indicated that oxidative stress was activated and cell proliferation was inhibited during transcriptional phase I, inflammation was widely activated during transcriptional phase II, muscle atrophy was activated and energy metabolism was significantly suppressed during transcriptional phase III, muscle atrophy and fibrosis was activated during transcriptional phase IV. Depending on these features, transcriptional phase I, II, III, and IV are, in sequence, designated as “oxidative stress stage,” “inflammation stage,” “atrophy stage,” and “atrophic fibrosis stage.”

At oxidative stress stage, cytochrome P450-related signaling pathways were remarkably activated. As is well known, cytochrome P450 enzymes catalyze the oxidative transformation of toxic metabolites into reactive oxygen species (ROS, such as hydroxyl radical and peroxide and superoxide anions), and mediate the mass production of ROS from estrogen metabolites (Khan et al., 2015; He L. et al., 2017), thereby becoming the marker of oxidative stress as well as the source of cellular ROS (Sato et al., 2011; Tomasi et al., 2018). On the other hand, monooxygenase activity, as a category of molecular function, was significantly enhanced also at oxidative stress stage. Actually, cytochrome P450 belongs to a class of monooxygenase enzymes, and is the main source of ROS due to its nature of terminal oxidases (Bhattacharyya et al., 2014). Our observations implied that a large amount of ROS was generated in response to oxidative stress during transcriptional phase I. Meanwhile, PPAR and HIF-1 signaling pathways were also activated at oxidative stress stage. PPARs (PPAR-α, -β/δ, and -γ), belonging to transcription factors, form a tightly connected triad, and they are involved in regulation of ROS production and degradation (Aleshin and Reiser, 2013; Morris et al., 2018). HIF-1, with a full name of hypoxia-inducible factor-1, plays a key role in genetic regulation of cellular adaptation to hypoxia. The excessive production of ROS under oxidative stress will activate HIF-1 signaling pathway to eliminate excess ROS and to prevent cellular damage (Jalouli et al., 2017; Eyrich et al., 2019). In a word, we found that oxidative stress was present at an early stage of denervated skeletal muscle atrophy. Although the involvement of oxidative stress in muscle atrophy (e.g., due to cancer cachexia or aging) has been reported (Faitg et al., 2017; Zhao et al., 2018), the genetic elucidation of oxidative stress impacts on denervated muscle atrophy, especially at the early stage, was really one of the highlights in this study. It is believable that this highlight may perhaps be extended to establishing an early antioxidant therapy to delay the progress of denervated skeletal muscle atrophy.

At inflammation stage, the up-regulated genes were related to activation of TNF, JAK-STAT, TGF-beta, NOD-like receptor, and NF-kappa B signaling pathways, as well as cytokine-cytokine receptor interaction, thus triggering a persistent inflammatory response (Philpott et al., 2014; Banerjee et al., 2017; He Y. et al., 2017). The inflammatory pathways are involved in different types of skeletal muscle atrophy caused by cachexia, aging, and/or chronic diseases (Perry et al., 2016; Zimmers et al., 2016; Sakurai and Harashima, 2017; Ma et al., 2018). STAT3 and NF-kappa B respond to the same upstream signal and cooperate to promote the expression of pro-cachectic genes, thus contributing to muscle atrophy (Ma et al., 2017). Interventions with JAK-STAT3 related inhibitors in a cancer cachexia model decrease IL-6 level and alleviate skeletal muscle atrophy (Bonetto et al., 2012). TGF-beta 1 mediates muscle atrophy (Zhu et al., 2017) and promotes fibroblast proliferation, collagen synthesis, and muscle fibrosis (Mendias et al., 2012). Moreover, proinflammatory cytokines (e.g., IL-6 and TNF-alpha) affect aging-induced skeletal muscle atrophy by regulating the related signaling pathways (Fan et al., 2016). Besides inflammatory signaling pathways, several biological processes, including immune response, inflammatory response, cellular response to IL-1 and cellular response to TNF, were also activated at inflammation stage. As to the enhanced molecular function at the same stage, they were CCR1 chemokine receptor binding, CCR2 chemokine receptor binding, CCR5 chemokine receptor binding, and cytokine activity. Our observation about the presence of inflammation stage not only provided new evidence for the significance of inflammation in skeletal muscle atrophy, but also inspired a new idea of using anti-inflammatory therapy for treating denervated skeletal muscle atrophy.

At atrophy stage, the signaling pathways related to the up-regulated genes were mainly phagosomes, lysosomes, endocytosis, and P53 signaling pathways, which were essential in proteolysis (Bonetto et al., 2012; Fanzani et al., 2012; Bonaldo and Sandri, 2013). There is overwhelming evidence that activation of autophagy/lysosomal and proteasome signaling pathways promotes protein degradation and causes muscle atrophy (Zhao et al., 2007). Further studies revealed that insulin activation inhibits the ubiquitin proteolytic system and the lysosomal hydrolysis system, promoting skeletal muscle hypertrophy or relieving skeletal muscle atrophy (O’Neill et al., 2016). Our results showed that the insulin signaling pathway was continuously inhibited to aggravate skeletal muscle atrophy. At atrophic fibrosis stage, the up-regulated genes were mainly involved in ribosomes, CAMs and ECM, phagosomes, and endocytosis. It has been reported that denervation for 14 days induces skeletal muscle fibrosis (Huang et al., 2016) and that synthesis of CAMs and ECM aggravates the fibrosis of injured skeletal muscle (Wynn and Ramalingam, 2012). Activation of ribosome-related signaling pathways might promote the synthesis of enzymes involved in the process of collagenation and fibrosis. These data suggested that at atrophic fibrosis stage, both the atrophic process (induced by protein proteolysis) and the fibrosis process (induced ECM overproduction) coexisted in denervated skeletal muscle.

At oxidative stress and inflammation stages, the down-regulated genes were involved in many molecular functions, including cell proliferation, cell cycle, DNA replication initiation, and mitotic sister chromatid segregation, indicating that cell proliferation was significantly inhibited at the two earlier stages of denervated skeletal muscle atrophy. At atrophy phase and atrophic fibrosis stages, the down-regulated genes were involved in many molecular functions, including cellular respiration, tricarboxylic acid cycle, fatty acid beta-oxidation, response to oxidative stress, and glycolytic process, indicating that the energy metabolism was significantly inhibited at the middle and later stages of denervated skeletal muscle atrophy. This phenomenon has also been noted in other models of skeletal muscle atrophy (Ibebunjo et al., 2013; Rao et al., 2018). It follows that the down-regulated genes during denervated skeletal muscle atrophy may be mainly involved in two molecular functions: cell proliferation and energy metabolism.

It should be mentioned that there are some limitations in this study. The rats used in the experiments grew considerably during the period of 0.25 h–28 days post nerve injury, and the response of skeletal muscle to denervation stimulus was accompanied by the body growth. It seems more suitable that the TA muscle samples harvested from the uninjured, contralateral side of animals at distinct times serve as multiple controls, which may be better than a single control (from sham-operated rats and designated as 0 h post surgery). Another drawback of our study was that lncRNA and microRNA were not investigated. Perhaps, the detection of mRNA expressions is not enough to support the whole transcriptome survey for obtaining more comprehensive information.

**FIGURE 9:**
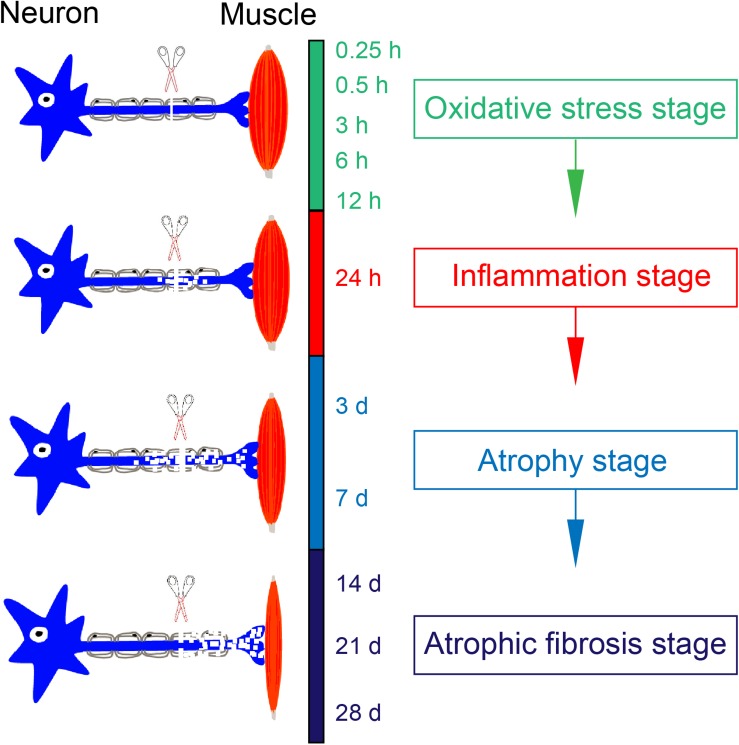
Schematic diagram illustrating a sequence of pathological processes in denervation-induced skeletal muscle atrophy. The disuse of skeletal muscle due to loss of innervation leads to ischemia and hypoxia of skeletal muscle, which contributes the production of ROS. This occurs at oxidative stress stage. A large amount of ROS may induce inflammation at inflammation stage. If inflammation is not controlled in time, it will activate the downstream proteolysis process, contributing to muscle atrophy. This occurs at atrophy stage and atrophic fibrosis stage.

In terms of four stages occurring within the period of 0.25 h–28 days post nerve injury, we assume that denervated skeletal muscle atrophy represents a cascade of gene expression changes, and it also consists of a sequence of pathological processes with each being triggered by a previous one. In brief, denervation (loss of innervations) causes the disuse of skeletal muscle, the disuse leads to ischemia and hypoxia for generating a large amount of ROS, ROS production initiates inflammation, and uncontrolled inflammation activates the downstream proteolysis process to induce skeletal muscle atrophy (see schematic in Figure 9). Overall, our results in this study may help understand the molecular mechanisms of denervated skeletal muscle atrophy and contribute to developing targeted therapeutic approaches.

## Data Availability Statement

The gene expression dataset is available on EBI and the accession number is E-MTAB-8009.

## Ethics Statement

The animal study was reviewed and approved by Nantong University Administration Committee of Experimental Animals, Jiangsu Province, China.

## Author Contributions

HS and JG designed the study. YS, RZ, LX, ZH, QW, WM, JZ, and MS performed the experiments. YS, ZH, WM, QW, JG, JZ, and LX collected and assembled the data. ZH, JZ, and WM performed the data analysis. FD provided the scientific expertise. HS wrote the manuscript.

## Conflict of Interest

The authors declare that the research was conducted in the absence of any commercial or financial relationships that could be construed as a potential conflict of interest.

## Footnotes

1 http://www.r-project.org

2 https://david.ncifcrf.gov

## Supplementary Material

TABLE S1 The primers used in the qPCR reactions are listed.

TABLE S2 Differentially expressed genes (DEGs) are identified from experimental groups compared to sham group.

## References

[B1] Aleshin S. Reiser G. (2013). Role of the peroxisome proliferator-activated receptors (PPAR)-alpha, beta/delta and gamma triad in regulation of reactive oxygen species signaling in brain. *Biol. Chem.* 394 1553–1570. 10.1515/hsz-2013-2215 24021597

[B2] Aversa Z. Zhang X. Fielding R. A. Lanza I. LeBrasseur N. K. (2019). The clinical impact and biological mechanisms of skeletal muscle aging. *Bone* 127 26–36. 10.1016/j.bone.2019.05.021 31128290 PMC6708726

[B3] Banerjee S. Biehl A. Gadina M. Hasni S. Schwartz D. M. (2017). Erratum to: JAK-STAT signaling as a target for inflammatory and autoimmune diseases: current and future prospects. *Drugs* 77 521–546. 10.1007/s40265-017-0772-777 28255960 PMC7102286

[B4] Bhattacharyya S. Sinha K. Sil P. C. (2014). Cytochrome P450s: mechanisms and biological implications in drug metabolism and its interaction with oxidative stress. *Curr. Drug Metab.* 15 719–742. 10.2174/1389200215666141125121659 25429675

[B5] Bilodeau P. A. Coyne E. S. Wing S. S. (2016). The ubiquitin proteasome system in atrophying skeletal muscle: roles and regulation. *Am. J. Physiol. Cell Physiol.* 311 C392–C403. 10.1152/ajpcell.00125.2016 27510905

[B6] Bodine S. C. Baehr L. M. (2014). Skeletal muscle atrophy and the E3 ubiquitin ligases MuRF1 and MAFbx/atrogin-1. *Am. J. Physiol. Endocrinol. Metab.* 307 E469–E484. 10.1152/ajpendo.00204.2014 25096180 PMC4166716

[B7] Bodine S. C. Latres E. Baumhueter S. Lai V. K. Nunez L. Clarke B. A. (2001). Identification of ubiquitin ligases required for skeletal muscle atrophy. *Science* 294 1704–1708. 10.1126/science.1065874 11679633

[B8] Bonaldo P. Sandri M. (2013). Cellular and molecular mechanisms of muscle atrophy. *Dis. Model Mech.* 6 25–39. 10.1242/dmm.010389 23268536 PMC3529336

[B9] Bonetto A. Aydogdu T. Jin X. Zhang Z. Zhan R. Puzis L. (2012). JAK/STAT3 pathway inhibition blocks skeletal muscle wasting downstream of IL-6 and in experimental cancer cachexia. *Am. J. Physiol. Endocrinol. Metab.* 303 E410–E421. 10.1152/ajpendo.00039.2012 22669242 PMC3423125

[B10] Chavez-Alvarez R. Chavoya A. Mendez-Vazquez A. (2014). Discovery of possible gene relationships through the application of self-organizing maps to DNA microarray databases. *PLoS One* 9:e93233. 10.1371/journal.pone.0093233 24699245 PMC3974722

[B11] Choi W. Lee J. Lee J. Ko K. R. Kim S. (2018). Hepatocyte growth factor regulates the miR-206-HDAC4 cascade to control neurogenic muscle atrophy following surgical denervation in mice. *Mol. Ther. Nucleic Acids* 12 568–577. 10.1016/j.omtn.2018.06.013 30195792 PMC6077135

[B12] Cohen S. Nathan J. A. Goldberg A. L. (2015). Muscle wasting in disease: molecular mechanisms and promising therapies. *Nat. Rev. Drug Discov.* 14 58–74. 10.1038/nrd4467 25549588

[B13] Eyrich N. W. Potts C. R. Robinson M. H. Maximov V. Kenney A. M. (2019). Reactive oxygen species signaling promotes hypoxia-inducible factor 1α stabilization in sonic hedgehog-driven cerebellar progenitor cell proliferation. *Mol. Cell. Biol.* 39:e00268-18. 10.1128/MCB.00268-18 PMC6447416 30692272

[B14] Faitg J. Reynaud O. Leduc-Gaudet J. P. Gouspillou G. (2017). Skeletal muscle aging and mitochondrial dysfunction: an update. *Med. Sci.* 33 955–962. 10.1051/medsci/20173311012 29200393

[B15] Fan J. Kou X. Yang Y. Chen N. (2016). MicroRNA-regulated proinflammatory cytokines in sarcopenia. *Mediators Inflamm.* 2016 1–9. 10.1155/2016/1438686 27382188 PMC4921629

[B16] Fanzani A. Conraads V. M. Penna F. Martinet W. (2012). Molecular and cellular mechanisms of skeletal muscle atrophy: an update. *J. Cachexia Sarcopenia Muscle* 3 163–179. 10.1007/s13539-012-0074-6 22673968 PMC3424188

[B17] Fedorov V. B. Goropashnaya A. V. Stewart N. C. Toien O. Chang C. Wang H. (2014). Comparative functional genomics of adaptation to muscular disuse in hibernating mammals. *Mol. Ecol.* 23 5524–5537. 10.1111/mec.12963 25314618 PMC4245363

[B18] Galili T. (2015). dendextend: an R package for visualizing, adjusting and comparing trees of hierarchical clustering. *Bioinformatics* 31 3718–3720. 10.1093/bioinformatics/btv428 26209431 PMC4817050

[B19] Glass D. Roubenoff R. (2010). Recent advances in the biology and therapy of muscle wasting. *Ann. N. Y. Acad. Sci.* 1211 25–36. 10.1111/j.1749-6632.2010.05809.x 21062293

[B20] Gomes M. D. Lecker S. H. Jagoe R. T. Navon A. Goldberg A. L. (2001). Atrogin-1, a muscle-specific F-box protein highly expressed during muscle atrophy. *Proc. Natl. Acad. Sci. U.S.A.* 98 14440–14445. 10.1073/pnas.251541198251541198 11717410 PMC64700

[B21] He L. He T. Farrar S. Ji L. Liu T. Ma X. (2017). Antioxidants maintain cellular redox homeostasis by elimination of reactive oxygen species. *Cell Physiol. Biochem.* 44 532–553. 10.1159/000485089 29145191

[B22] He Y. Xiao Y. Yang X. Li Y. Wang B. Yao F. (2017). SIRT6 inhibits TNF-alpha-induced inflammation of vascular adventitial fibroblasts through ROS and Akt signaling pathway. *Exp. Cell Res.* 357 88–97. 10.1016/j.yexcr.2017.05.001 28477980

[B23] He Q. Qiu J. Dai M. Fang Q. Sun X. Gong Y. (2016). MicroRNA-351 inhibits denervation-induced muscle atrophy by targeting TRAF6. *Exp. Ther. Med.* 12 4029–4034. 10.3892/etm.2016.3856 28101181 PMC5228305

[B24] Huang Q. K. Qiao H. Y. Fu M. H. Li G. Li W. B. Chen Z. (2016). Mir-206 attenuates denervation-induced skeletal muscle atrophy in rats through regulation of satellite cell differentiation via tgf-beta1, smad3, and hdac4 signaling. *Med. Sci. Monit.* 22 1161–1170. 10.12659/msm.897909 27054781 PMC4829125

[B25] Ibebunjo C. Chick J. M. Kendall T. Eash J. K. Li C. Zhang Y. (2013). Genomic and proteomic profiling reveals reduced mitochondrial function and disruption of the neuromuscular junction driving rat sarcopenia. *Mol. Cell. Biol.* 33 194–212. 10.1128/MCB.01036-1012 23109432 PMC3554128

[B26] Jalouli M. Mokas S. Turgeon C. A. Lamalice L. Richard D. E. (2017). Selective HIF-1 regulation under nonhypoxic conditions by the p42/p44 MAP kinase inhibitor PD184161. *Mol. Pharmacol.* 92 510–518. 10.1124/mol.117.108654 28814529

[B27] Jeong H. O. Park D. Im E. Lee J. Im D. S. Chung H. Y. (2017). Determination of the mechanisms that cause sarcopenia through cDNA microarray. *J. Frailty Aging* 6 97–102. 10.14283/jfa.2017.13 28555711

[B28] Khan M. A. Zubair H. Srivastava S. K. Singh S. Singh A. P. (2015). Insights into the role of microRNAs in pancreatic cancer pathogenesis: potential for diagnosis, prognosis, and therapy. *Adv. Exp. Med. Biol.* 889 71–87. 10.1007/978-3-319-23730-5_5 26658997 PMC5706654

[B29] Khatri P. Sirota M. Butte A. J. (2012). Ten years of pathway analysis: current approaches and outstanding challenges. *PLoS Comput. Biol.* 8:e1002375. 10.1371/journal.pcbi.1002375 22383865 PMC3285573

[B30] Kolde R. (2015). *Package ‘Pheatmap’.* R package version 1.0. 8.

[B31] Kruger K. Dischereit G. Seimetz M. Wilhelm J. Weissmann N. Mooren F. C. (2015). Time course of cigarette smoke-induced changes of systemic inflammation and muscle structure. *Am. J. Physiol. Lung Cell Mol. Physiol.* 309 L119–L128. 10.1152/ajplung.00074.2015 26001775

[B32] Kunkel S. D. Suneja M. Ebert S. M. Bongers K. S. Fox D. K. Malmberg S. E. (2011). mRNA expression signatures of human skeletal muscle atrophy identify a natural compound that increases muscle mass. *Cell Metab.* 13 627–638. 10.1016/j.cmet.2011.03.020 21641545 PMC3120768

[B33] Lala-Tabbert N. Lejmi-Mrad R. Timusk K. Fukano M. Holbrook J. St-Jean M. (2019). Targeted ablation of the cellular inhibitor of apoptosis 1 (cIAP1) attenuates denervation-induced skeletal muscle atrophy. *Skelet Muscle* 9:13. 10.1186/s13395-019-0201-206 31126323 PMC6533726

[B34] Laughlin M. H. (1987). Skeletal muscle blood flow capacity: role of muscle pump in exercise hyperemia. *Am. J. Physiol.* 253(5 Pt 2), H993–H1004. 10.1152/ajpheart.1987.253.5.H993 3318504

[B35] Lever J. Krzywinski M. Altman N. (2017). Principal component analysis. *Nat. Methods* 14:641 10.1038/nmeth.4346

[B36] Li J. Chan M. C. Yu Y. Bei Y. Chen P. Zhou Q. (2017). miR-29b contributes to multiple types of muscle atrophy. *Nat. Commun.* 8:15201. 10.1038/ncomms15201 28541289 PMC5458521

[B37] Livak K. J. Schmittgen T. D. (2001). Analysis of relative gene expression data using real-time quantitative PCR and the 2(-Delta Delta C(T)) Method. *Methods* 25 402–408. 10.1006/meth.2001.1262 11846609

[B38] Ma J. F. Sanchez B. J. Hall D. T. Tremblay A. K. Di Marco S. Gallouzi I. E. (2017). STAT3 promotes IFNgamma/TNFalpha-induced muscle wasting in an NF-kappaB-dependent and IL-6-independent manner. *EMBO Mol. Med.* 9 622–637. 10.15252/emmm.201607052 28264935 PMC5412921

[B39] Ma W. Xu T. Wang Y. Wu C. Wang L. Yang X. (2018). The role of inflammatory factors in skeletal muscle injury. *Biotarget* 2:7 10.21037/biotarget.2018.04.01

[B40] Madaro L. Passafaro M. Sala D. Etxaniz U. Lugarini F. Proietti D. (2018). Denervation-activated STAT3-IL-6 signalling in fibro-adipogenic progenitors promotes myofibres atrophy and fibrosis. *Nat. Cell Biol.* 20 917–927. 10.1038/s41556-018-0151-y 30050118 PMC6145844

[B41] Marra A. M. Arcopinto M. Bossone E. Ehlken N. Cittadini A. Grunig E. (2015). Pulmonary arterial hypertension-related myopathy: an overview of current data and future perspectives. *Nutr. Metab. Cardiovasc. Dis.* 25 131–139. 10.1016/j.numecd.2014.10.005 25455722

[B42] Mendias C. L. Gumucio J. P. Davis M. E. Bromley C. W. Davis C. S. Brooks S. V. (2012). Transforming growth factor-beta induces skeletal muscle atrophy and fibrosis through the induction of atrogin-1 and scleraxis. *Muscle Nerve* 45 55–59. 10.1002/mus.22232 22190307 PMC3245632

[B43] Morris C. J. Kameny R. J. Boehme J. Gong W. He Y. Zhu T. (2018). KLF2-mediated disruption of PPAR-gamma signaling in lymphatic endothelial cells exposed to chronically increased pulmonary lymph flow. *Am. J. Physiol. Heart Circ. Physiol.* 315 H173–H181. 10.1152/ajpheart.00635.2017 29631374 PMC6087778

[B44] O’Neill B. T. Lee K. Y. Klaus K. Softic S. Krumpoch M. T. Fentz J. (2016). Insulin and IGF-1 receptors regulate FoxO-mediated signaling in muscle proteostasis. *J. Clin. Invest.* 126 3433–3446. 10.1172/jci86522 27525440 PMC5004956

[B45] Oost L. J. Kustermann M. Armani A. Blaauw B. Romanello V. (2019). Fibroblast growth factor 21 controls mitophagy and muscle mass. *J Cachexia Sarcopenia Muscle* 10 630–642. 10.1002/jcsm.12409 30895728 PMC6596457

[B46] Parikh J. R. Klinger B. Xia Y. Marto J. A. Bluthgen N. (2010). Discovering causal signaling pathways through gene-expression patterns. *Nucleic Acids Res.* 38 W109–W117. 10.1093/nar/gkq424 20494976 PMC2896193

[B47] Perry B. D. Caldow M. K. Brennan-Speranza T. C. Sbaraglia M. Jerums G. Garnham A. (2016). Muscle atrophy in patients with Type 2 Diabetes Mellitus: roles of inflammatory pathways, physical activity and exercise. *Exerc. Immunol. Rev.* 22 94–109. 26859514 PMC5545118

[B48] Philpott D. J. Sorbara M. T. Robertson S. J. Croitoru K. Girardin S. E. (2014). NOD proteins: regulators of inflammation in health and disease. *Nat. Rev. Immunol.* 14 9–23. 10.1038/nri3565 24336102

[B49] Pigna E. Renzini A. Greco E. Simonazzi E. Fulle S. Mancinelli R. (2018). HDAC4 preserves skeletal muscle structure following long-term denervation by mediating distinct cellular responses. *Skelet Muscle* 8:6. 10.1186/s13395-018-0153-152 29477142 PMC6389241

[B50] Qiu J. Fang Q. Xu T. Wu C. Xu L. Wang L. (2018). Mechanistic role of reactive oxygen species and therapeutic potential of antioxidants in denervation- or fasting-induced skeletal muscle atrophy. *Front. Physiol.* 9:215. 10.3389/fphys.2018.00215 29593571 PMC5861206

[B51] Rao M. Jaber B. L. Balakrishnan V. S. (2018). Chronic kidney disease and acquired mitochondrial myopathy. *Curr. Opin. Nephrol. Hypertens* 27 113–120. 10.1097/MNH.0000000000000393 29266014

[B52] Sacheck J. M. Hyatt J. P. Raffaello A. Jagoe R. T. Roy R. R. Edgerton V. R. (2007). Rapid disuse and denervation atrophy involve transcriptional changes similar to those of muscle wasting during systemic diseases. *FASEB J.* 21 140–155. 10.1096/fj.06-6604com 17116744

[B53] Sakurai Y. Harashima H. (2017). A complicated interpretation of a therapeutic effect with humanized mice using a novel peptide platform. *Biotarget* 1:4 10.21037/biotarget.2017.05.04

[B54] Sato M. Yokoyama U. Fujita T. Okumura S. Ishikawa Y. (2011). The roles of cytochrome p450 in ischemic heart disease. *Curr. Drug Metab.* 12 526–532. 10.2174/138920011795713715 21476972

[B55] Szentesi P. Csernoch L. Dux L. Keller-Pintér A. (2019). Changes in redox signaling in the skeletal muscle with aging. *Oxid. Med. Cell Longev.* 2019:4617801. 10.1155/2019/4617801 30800208 PMC6360032

[B56] Tang H. Inoki K. Lee M. Wright E. Khuong A. Khuong A. (2014). mTORC1 promotes denervation-induced muscle atrophy through a mechanism involving the activation of FoxO and E3 ubiquitin ligases. *Sci. Signal.* 7:ra18. 10.1126/scisignal.2004809 24570486

[B57] Tomasi M. L. Ramani K. Ryoo M. Cossu C. Floris A. Murray B. J. (2018). SUMOylation regulates cytochrome P450 2E1 expression and activity in alcoholic liver disease. *FASEB J.* 32 3278–3288. 10.1096/fj.201701124R 29401608 PMC5956242

[B58] Weng J. Zhang P. Yin X. Jiang B. (2018). The whole transcriptome involved in denervated muscle atrophy following peripheral nerve injury. *Front. Mol. Neurosci.* 11:69. 10.3389/fnmol.2018.00069 29563865 PMC5845901

[B59] Wu C. L. Cornwell E. W. Jackman R. W. Kandarian S. C. (2014). NF-kappaB but not FoxO sites in the MuRF1 promoter are required for transcriptional activation in disuse muscle atrophy. *Am. J. Physiol. Cell Physiol.* 306 C762–C767. 10.1152/ajpcell.00361.2013 24553183 PMC3989716

[B60] Wynn T. A. Ramalingam T. R. (2012). Mechanisms of fibrosis: therapeutic translation for fibrotic disease. *Nat. Med.* 18 1028–1040. 10.1038/nm.2807 22772564 PMC3405917

[B61] Zhao J. Brault J. J. Schild A. Cao P. Sandri M. Schiaffino S. (2007). FoxO3 coordinately activates protein degradation by the autophagic/lysosomal and proteasomal pathways in atrophying muscle cells. *Cell Metab.* 6 472–483. 10.1016/j.cmet.2007.11.004 18054316

[B62] Zhao J. Tian Z. Kadomatsu T. Xie P. Miyata K. Sugizaki T. (2018). Age-dependent increase in angiopoietin-like protein 2 accelerates skeletal muscle loss in mice. *J. Biol. Chem.* 293 1596–1609. 10.1074/jbc.M117.814996 29191837 PMC5798292

[B63] Zhu X. Kny M. Schmidt F. Hahn A. Wollersheim T. Kleber C. (2017). Secreted frizzled-related protein 2 and inflammation-induced skeletal muscle atrophy. *Crit. Care Med.* 45 e169–e183. 10.1097/CCM.0000000000002056 27661566

[B64] Zimmers T. A. Fishel M. L. Bonetto A. (2016). STAT3 in the systemic inflammation of cancer cachexia. *Semin. Cell Dev. Biol.* 54 28–41. 10.1016/j.semcdb.2016.02.009 26860754 PMC4867234

